# An Improved YOLO11n-Based Algorithm for Road Sign Detection

**DOI:** 10.3390/s26082543

**Published:** 2026-04-20

**Authors:** Haifeng Fu, Xinlei Xiao, Yonghua Han, Le Dai, Lan Yao, Lu Xu

**Affiliations:** School of Information Science and Engineering, Zhejiang Sci-Tech University, Hangzhou 310018, China; 2023332871016@mails.zstu.edu.cn (H.F.); 2024331200046@mails.zstu.edu.cn (X.X.); 2023329600141@mails.zstu.edu.cn (L.D.); 2023331200034@mails.zstu.edu.cn (L.Y.); xulu@zstu.edu.cn (L.X.)

**Keywords:** road sign detection, improved YOLO11n network, MGA module, neck, improved SPPF module

## Abstract

**Highlights:**

**What are the main findings?**

- To improve the performance of road sign detection algorithms in complex backgrounds for detecting multi-scale, low-resolution, and occluded small targets, a Multi-path Gated Aggregation module is designed. This module consists of a multi-scale feature extraction branch, a color feature extraction branch, and a detail feature extraction branch.
- In order to enhance the detection performance of the YOLO11n algorithm for small and blurry targets, high-resolution information from the shallow layers of the Backbone is integrated into the Neck network. Moreover, a Group Convolution-Layer Normalization-SiLU (GLS) structure is designed in the SPPF structure to achieve the fusion of information at different levels during SPPF information transmission.

**What are the implications of the main findings?**

- A modular design paradigm is proposed for the feature extraction from multi-scale, low-resolution, and small targets in complex backgrounds. The introduced channel interaction mechanism and GLS-enhanced SPPF structure provide reusable structural references and design insights for improving the robustness of target detection under occlusion, blurring, low-resolution, and multi-scale variation in complex scenes.
- It attains a high balance between detection accuracy and real-time performance, making it suitable for vehicle deployment. While maintaining a high frame rate of 134 FPS, this method significantly improves the detection accuracy of various road signs (especially for low-resolution, partially occluded, and small targets) and can meet the dual requirements of reliability and real-time performance of intelligent driving systems in real and complex scenes.

**Abstract:**

For vehicle driving scenarios in complex backgrounds, road sign detection faces challenges such as multi-scale targets, long-distances, and low-resolution. To address these challenges, a detection method based on an improved YOLO11n network is proposed. Firstly, to accommodate the multi-scale characteristics of the targets and improve the network’s ability to detect low-resolution objects and details, a Multi-path Gated Aggregation (MGA) Module is proposed, achieving these objectives via multi-dimensional feature extraction. Secondly, the Neck is improved by designing a network structure that incorporates high-resolution information from the Backbone, thereby enhancing the detection capabilities for small and blurry targets. Finally, an enhanced Spatial Pyramid Pooling—Fast (SPPF) module is proposed, wherein a Group Convolution-Layer Normalization-SiLU structure is integrated across various stages of information passing. By fusing adjacent channel information, it effectively suppresses complex background noise across multiple scales and amplifies road marking features, which consequently boosts the model’s discriminability for distant and obscured targets. Experimental results on a multi-type road sign dataset show that the improved model achieves an mAP@0.5 of 96.96%, which is 1.42% higher than the original model. The mAP@0.5–0.95 and Recall rates are 83.94% and 92.94%, respectively, while the inference speed remains at 134 FPS. Research demonstrates that via targeted modular designs, the proposed approach strikes a superior balance between detection accuracy and real-time efficiency. Consequently, it provides robust technical support for the reliable operation of intelligent vehicle perception systems under complex conditions.

## 1. Introduction

With the development of artificial intelligence and visual inspection technologies, autonomous driving systems are facing increasingly stringent safety requirements [1]. In this context, accurate road sign detection has become a crucial safeguard for driving safety [2].

The traditional algorithms for detecting road signs are mainly divided into three categories: The first category is based on color features. Representative methods include template matching and threshold segmentation in the RGB color space [3], road sign color extraction combined with denoising [4], and clustering analysis based on fused RGB and HIS color-space features [5]. However, such methods are greatly affected by environmental interference and lack robustness. The second category relies on shape features. Representative approaches include extracting line elements with the Hough transform to recognize specific signs [6] and constructing radial symmetry detection models for circular sign recognition [7]. In addition, similarity detection [8], distance transform matching [9], edge detection [10], and Haar-like feature detection [11] have also been widely used. Nevertheless, these methods are sensitive to sign occlusion and physical deformation, such as environmental erosion, which limits their overall robustness. The third category focuses on the combination of color and shape features. One approach achieves fine-grained morphological classification of candidate regions by integrating color saturation with the support vector machine with an integrated radial basis function (RBF) kernel [12]. Another method improves the robustness of circular sign detection through color segmentation, edge filtering, and Random Sample Consensus (RANSAC) ellipse fitting [13]. Although dual-feature fusion enhances detection precision, these models remain sensitive to ambient color interference. They are also vulnerable to challenges such as road sign occlusion and deformation.

In conclusion, despite the continuous iterative refinements of conventional algorithms, they remain constrained by limited environmental robustness and suboptimal detection precision.

Deep learning possesses capabilities for automatic feature extraction and generalization, enabling it to overcome the dependence on environment and shape in traditional methods. To address the real-time and multi-scale challenges in traffic sign detection, an improved model combining scale transformation with extreme learning machine [14] and a region proposal network based on Faster R-CNN [15] have been proposed. To address challenges such as small-object detection, researchers have proposed the cross-receptive-field RFB-c model [16] and a traffic sign detection approach based on RetinaNet-NeXt [17]. Although these methods have achieved improvements in detection accuracy, they fail to consider scale variations among traffic signs in the design of the detection heads, making it difficult to simultaneously balance detection accuracy for multi-scale targets and inference efficiency. In addition, the model size is too large, which is not conducive to local deployment.

Therefore, YOLO series multi-head detection structures with multi-scale detection capabilities have gradually become widely used. For instance, YOLO3 was employed on the GTSDB traffic sign dataset [18], whereas a GhostNet-enhanced YOLO4 was developed for real-time traffic sign detection under limited computational budgets [19]. These early YOLO versions were developed within the traditional Convolutional Neural Network (CNN) algorithms. Their feature extraction was constrained by local receptive fields. As a result, they still showed clear limitations in global contextual interaction, efficient multi-scale feature fusion, and precise perception of small objects. Further improvements are therefore still needed.

To address these limitations, subsequent studies have introduced further improvements. For instance, the CSW-YOLO model was proposed, which accelerates bounding box regression by adopting the Wise-Inner-MPDIoU loss function, thereby effectively enhancing the detection performance of small-scale traffic signs in complex traffic scenes [20]. Similarly, the DP-YOLO model was developed, which improves the feature representation capability of small objects while reducing model complexity through the reconstruction of the detection hierarchy and the integration of CNN and Transformer modules [21]. These efforts indicate that current object detection methods are evolving toward stronger small-object perception, more lightweight architectures, and greater robustness in complex environments. Nevertheless, for traffic sign detection tasks involving low resolution, occlusion, and complex backgrounds, further improvements are still needed.

Overall, existing studies have made progress in network architecture optimization and real-time performance improvement. However, they still suffer from limitations in model lightweight design and generalization in complex scenarios. Notably, detection performance significantly degrades under challenging conditions such as small-scale road signs, occlusion, backlighting, and long distances. Accordingly, there is an urgent need for an approach that balances detection accuracy and stability to enhance the model’s capability to detect road signs under diverse conditions.

To address these issues, a multi-scale road sign detection method based on an improved YOLO11n model is proposed. The improved model incorporates newly designed feature extraction modules and an optimized neck structure, significantly bolstering detection accuracy while maintaining real-time performance, particularly excelling in long-range and small-object recognition.

The main contributions are summarized as follows:

1. To address the detection challenges of low-resolution and occluded targets in complex backgrounds, an MGA module is proposed. The module comprises two core components: multi-path feature extraction and channel semantic interaction. The multi-path feature extraction branch consists of three parallel paths, comprising a 1 × 1 convolutional layer, a 3 × 3 convolutional layer, and a 5 × 5 Depth-Wise Separable Convolution (DWConv) for context capture. These paths extract road sign features from small, medium, and large receptive fields. The channel semantic interaction branch first divides the input features into two paths along the channel dimension. The channel elements of the two paths are then multiplied in an element-wise manner to strengthen cross-channel semantic correlations. In parallel, a 1 × 1 convolution branch is employed to preserve detailed information. This design significantly enhances the model’s ability to detect low-resolution and occluded small traffic signs in complex scenarios, and provides a structural reference for addressing other challenging detection tasks.
2. To address the challenges of small-scale object detection, an improved neck architecture is proposed based on YOLO11n. The improved neck structure introduces a feature fusion path based on max-pooling downsampling. A branch is extended from the high-resolution feature layer of the YOLO11n backbone. A max-pooling operation with a stride of 2 is applied to adjust the spatial resolution of the feature map. The resulting feature map is then introduced into the neck for subsequent feature fusion. The max-pooling operation preserves fine-grained detail information. After concatenation and fusion with the multi-scale features in the neck, the resulting features improve the detail representation capability of high-level semantic features without introducing additional parameters. This design thus mitigates the loss of small-scale traffic sign features in high-level feature maps. This neck network provides an effective design for multi-scale traffic sign feature fusion.
3. To address the detection of long-range and blurry targets, an improved architecture based on SPPF is proposed. Following the max-pooling operation in the SPPF module, a Group Convolution-Layer Normalization-SiLU (GLS) module is introduced to alleviate the relative sparsity of traffic sign features. Its main objective is to enhance sparse representations within multi-scale feature layers via adjacent channel fusion, simultaneously preserving the distributional diversity of features across various channels. Ultimately, with the integration of the improved SPPF, the information flow becomes richer and smoother, effectively strengthening the model’s perception capability for distant imaging and blurred signs. This structure offers a valuable design reference for distant target detection tasks.

## 2. Proposed Methods

### 2.1. YOLO11n Algorithm Architecture

#### 2.1.1. Introduction to the YOLO11n Algorithm

**Figure 1:**
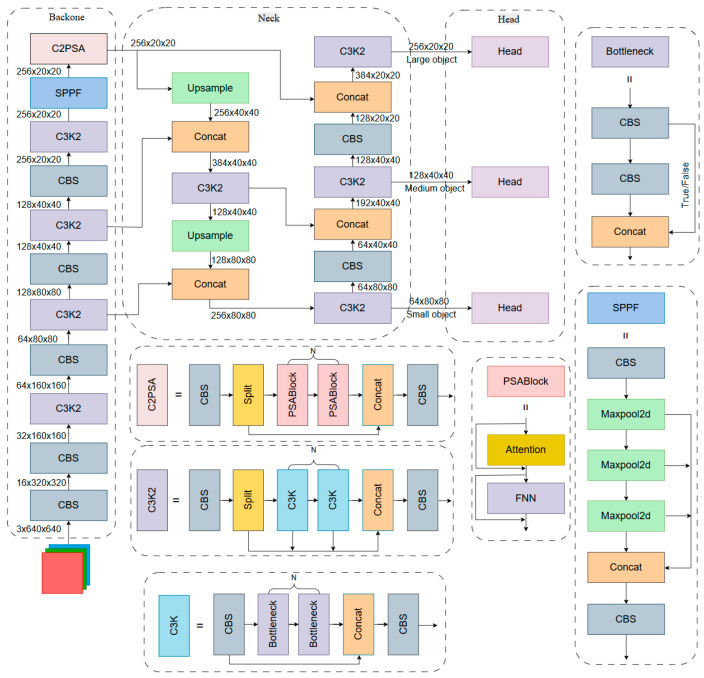
YOLO11n network architecture diagram.

In 2024, the Ultralytics team launched YOLO11n, a lightweight object detection model [22]. As the successor to YOLO8, YOLO11n inherits the overall design paradigm of YOLO8. However, it replaces the C2f structure used in YOLO8 with the C3k2 module in the backbone [23] and further introduces the C2PSA spatial attention mechanism. These improvements make it more suitable for high-accuracy and real-time object detection in complex backgrounds. The network architecture of YOLO11n is shown in Figure 1.

Figure 1 illustrates the network structure of YOLO11n, which consists of three core modules: the Backbone, the Neck, and the Head. The C3k2 module in the backbone network not only possesses the flexibility of the CSP Stage Block with 3 Convolutions and 2-input Bottleneck (C3k) module but also inherits the high-speed advantage of C2f, enabling superior feature reuse and gradient propagation with lower computational cost. The built-in Split structure in the C3K2 module splits the input features along the channel dimension. One branch acts as a direct shortcut, while the other is processed for feature extraction, allowing for feature reuse with low computational overhead. In the Backbone, multiple Convolution (Conv) modules and C3K2 modules are stacked sequentially to extract both shallow-level spatial features and deep-level semantic features from the input images. At the end of the Backbone, the model introduces the SPPF module to aggregate global information. Concurrently, the Cross Stage Partial with Pyramid Squeeze Attention (C2PSA) module is integrated after the SPPF module, utilizing a self-attention mechanism to enhance the model’s target feature extraction capability.

In the Neck network, YOLO11n adopts an enhanced bidirectional feature fusion scheme based on the Path Aggregation Network-Feature Pyramid Network (PAN-FPN) [24], achieving a more lightweight design and improved fusion efficiency. Through the combination of multi-layer upsampling, feature concatenation and C3K2 integration blocks, this section enables cross-scale information interaction. The optimized Neck exhibits stronger feature extraction capability when dealing with complex backgrounds and small-scale targets. It maintains high-quality semantic fusion while effectively reducing computational redundancy.

In the final Head portion, YOLO11n introduces DWConv to replace the traditional Conv in YOLO8. This approach substantially cuts the computational load without compromising feature representation ability, better accommodating the real-time requirements for multi-scale object detection [25].

#### 2.1.2. Analysis of the YOLO11n Algorithm

YOLO11n’s Backbone network first receives an input image with 3 channels and a spatial resolution of 640 × 640 pixels. Through consecutive Convolution-Batch Normalization-SiLU (CBS) and C3K2 modules, it performs multi-layer feature extraction and downsampling. With the increase in network depth, the spatial dimensions are progressively downsampled from 320 × 320 and 160 × 160 to 80 × 80, 40 × 40, and, finally, 20 × 20, while the channel dimension is expanded to 256. Through this process, the model achieves effective extraction and fusion of the detection targets, transitioning from shallow local textures to global semantic information. In real-world road sign detection, signs often vary markedly in shape and orientation. The C3k2 module combines the flexibility of the C3k structure with the lightweight efficiency of C2f, and therefore offers clear advantages in capturing sign features under diverse poses and scales [26].

As shown in Figure 1, the SPPF module, introduced at the end of the Backbone network, progressively enlarges the receptive field via multi-scale pooling to consolidate context information. The integration of the SPPF module enables the model to effectively handle distant small-sized signs or occlusion scenarios while mitigating interference caused by complex backgrounds [27]. The subsequent C2PSA module incorporates a self-attention mechanism, allowing the model to adaptively allocate feature weights across both the spatial and channel dimensions. Consequently, this guides the model to spontaneously attend to the highly discriminative shape and color features of the targets [28].

Building upon the PAN-FPN framework, the Neck network utilizes a lightweight bidirectional fusion mechanism. It integrates deep semantics into shallow layers and passes low-level spatial details upwards, enabling the joint optimization of spatial localization and semantic information. This bidirectional feature fusion strategy improves the model’s detection capability for small-sized signs [24].

Based on the preceding analysis, the YOLO11 structure is well-suited for road sign detection tasks. However, it still faces several challenges: its attentional focus on low-resolution, occluded, and small-scale signs under complex backgrounds is inadequate; furthermore, the robustness of the model degrades under distant, backlit, and obscure conditions. These limitations restrict YOLO11n from handling more challenging road sign detection tasks.

To solve these challenges and further improve the accuracy and robustness of the model, several modifications were made to YOLO11n. In particular, an MGA feature extraction module was designed to replace the C3k2 module in the backbone. In addition, the original neck network was optimized, and the SPPF module was further improved.

### 2.2. Improved YOLO11n Algorithm

**Figure 2:**
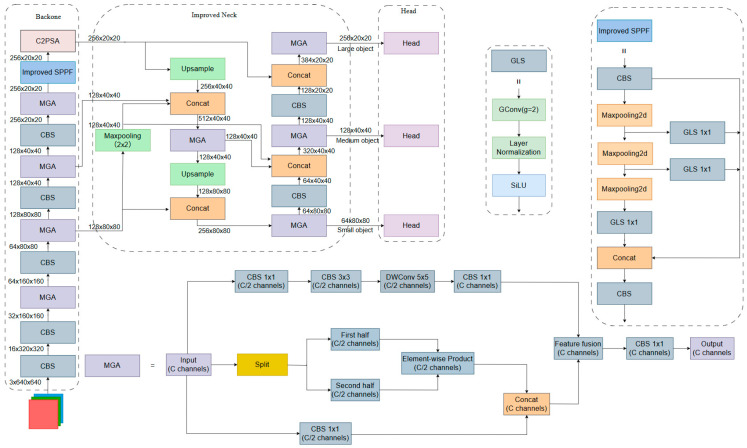
Improved YOLO11n network architecture diagram.

To address the detection challenges mentioned above, improvements are made to the YOLO11n model, constructing an accurate and robust road sign detection model, as shown in Figure 2.

Firstly, the MGA module was designed. The module introduces three parallel branches: multi-scale feature enhancement, color feature interaction, and edge detail preservation. Without significantly increasing the computational burden, the MGA module enhances the model’s ability to represent road sign features under low-resolution, complex background, and occlusion scenarios. As a result, it strengthens detection robustness.

Secondly, a max-pooling branch was introduced into the neck, originating from the high-resolution feature layer of the Backbone. Pooling-based downsampling provides a strong feature preservation capability. It can effectively alleviate the problem where small-scale target features are prone to being lost during feature extraction and improve the model’s detection performance for small targets in complex scenes.

Finally, the SPPF module is improved. The improved SPPF module sequentially embeds Group Convolution, Layer Normalization, and SiLU activation functions after multi-level max-pooling downsampling output, referred to as the GLS structure. The GLS structure progressively normalizes and stabilizes feature distributions across different scales, thereby effectively mitigating the feature distribution shift caused by scale differences. Benefiting from this stable representation of multi-scale features, the improved SPPF module further enhances the model’s feature aggregation and representation capabilities for distant and blurred road signs.

#### 2.2.1. The Multi-Path Gated Aggregation Module

In the C3K2 module of YOLO11n, shown in Figure 1, the Split module divides the input feature map by channel. One part undergoes feature fusion within the C3K module, while the other serves as a detail layer, bypassing directly to the ensuing Concat layer. However, the detailed features directly forwarded to the Concat layer contain only half of the feature channels. In vehicle driving scenarios, when road signs have low resolution, the loss of detailed information may lead to insufficient representation of the fine-grained features of road signs, thereby degrading the detection capability for small or blurred signs.

Furthermore, the feature extraction capability of the C3K2 module in YOLO11n mainly relies on two sequentially stacked Bottleneck layers. This structure leads to a restricted receptive field. As a result, YOLO11n is limited in its capacity to capture the structural features of large-scale traffic signs in the input image, which poses challenges for high-precision detection.

**Figure 3:**
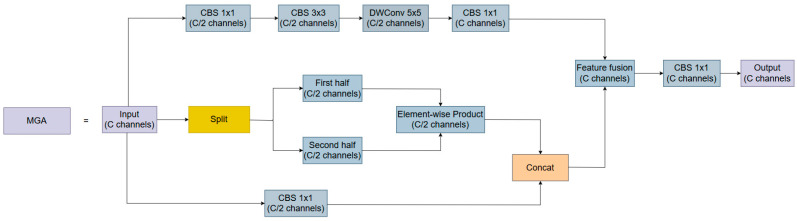
The structure of the Multi-path Gated Aggregation (MGA) module.

In summary, in driving scenarios, the Split module of YOLO11n shows limited capability in extracting fine-grained features, while the C3K2 module is less effective at enhancing large-scale information. As a result, YOLO11n exhibits weaker detection performance under scale variation, complex backgrounds, low-resolution conditions, and occlusion. This increases the risk of false and missed detections. To address the above issues, the MGA module illustrated in Figure 3 is proposed.

The MGA module comprises three core components: a receptive-field enhancement branch for multi-scale object detection, a color feature extraction branch for addressing complex backgrounds, low resolution, and occlusion, and a detail feature extraction branch. The three branches are further integrated through channel concatenation and channel-wise feature fusion, thereby improving the discriminability and robustness of the module’s feature representations.

As illustrated in the top section of Figure 3, this part corresponds to the multi-scale receptive-field enhancement branch, which is designed to handle the substantial scale variation of traffic signs in imaging. Compared with the conventional strategy of directly stacking multiple standard CBS convolutional layers to enlarge the receptive field in traditional networks [29], this branch enables more efficient lightweight feature extraction through four cascaded units.

For the design of the multi-scale receptive-field enhancement branch, the sparsity of traffic sign features and the resulting tendency of features across channels to become similar were taken into account [30]. The input channels are first compressed from C to C/2 through a 1 × 1 CBS convolution. This design facilitates cross-channel information interaction while reducing the computational burden of subsequent operations. Subsequently, a 3 × 3 standard CBS convolution is employed to facilitate sufficient fusion of features with a relatively small receptive field, thereby supporting the accurate localization of medium-sized targets. Next, to avoid the sharp increase in parameters caused by stacking consecutive standard convolutions, a 5 × 5 depthwise separable convolution is incorporated. This design efficiently enlarges the receptive field while maintaining extremely low computational cost, and thus helps capture the coarse contextual information of large targets. Finally, the aforementioned 5 × 5 depthwise separable convolution suffers from insufficient channel information fusion, which may further aggravate the sparsity and similarity of channel-wise traffic sign features and thus impair feature extraction. To overcome this limitation, a 1 × 1 CBS convolution is introduced again to achieve deep fusion of multi-scale spatial features and channel features. This design aims to extract local details and contextual information without introducing notable computational overhead, thereby enabling high-quality feature representation.

As shown in Figure 3, the middle branch is the color feature extraction branch, which is used to extract the color features of road signs. Introducing color features improves the detection rate of low-resolution and occluded road signs under complex backgrounds. This branch first divides the input feature map into two sub-feature maps $X_{1},X_{2}\in {\mathbb{R}}^{H\times W\times C/2}$. Then, as shown in Equation (1) below, an element-wise product operation is performed on the corresponding channels of the two sub-feature maps.

(1) $$X_{\mathrm{int}}=X_{1}⊙X_{2}$$

where ⊙ denotes element-wise multiplication.

The primary consideration is that the color features of road signs typically exhibit regular shapes, representing large-scale information compared to local details such as edges. Splitting features of the same scale along the channel dimension and performing element-wise multiplication can amplify the differences in feature values [31]. This operation highlights road sign features with large scales and relatively uniform colors, while suppressing inconsistent redundancy or noise, thereby enhancing the discriminability of feature representation. In contrast to local details, such large-scale features exhibit greater robustness against low-resolution and occlusion. Therefore, this structure assists the model in effectively separating road markings from the background, thereby reducing the rates of missed and false detections.

As shown by the lower structure in Figure 3, this part corresponds to the detail feature extraction branch, which is designed to preserve fine-grained information such as edges of traffic signs. This helps improve the detection rate of traffic signs in complex backgrounds. Road signs exhibit distinct shape characteristics and relatively uniform colors, which can be effectively captured by edge information. Therefore, this branch processes the C-channel features of the corresponding scale through a 1 × 1 CBS convolution to achieve channel fusion and enhance edge details. The CBS feature extraction module effectively alleviates the loss of fine details that commonly occurs in conventional networks. By virtue of its smooth and non-monotonic mathematical properties, the built-in SiLU activation function avoids truncating subtle negative-gradient information in shallow convolutions. This theoretically supports the preservation and enhanced representation of weak detail features, such as edges and textures [32]. Considering the sparsity and similarity of channel-wise features, and to facilitate subsequent operations while preserving a lightweight design, a CBS operation is first applied to reduce the channel dimension from C to C/2. The output of the color feature extraction branch is then concatenated with that of the detail feature extraction branch, generating an enhanced C-channel feature map with both color and edge awareness. This component is defined as the channel semantic interaction component.

As shown in Figure 3, feature aggregation is performed at the final stage of feature integration by the Feature Fusion operator. It adds the C-channel output of the multi-scale receptive-field enhancement branch to the C-channel output of the channel semantic interaction component in a channel-wise manner. Subsequently, a 1 × 1 CBS convolution is applied to ensure deep fusion of multi-dimensional feature information. This fusion combines multi-scale information with road sign cues such as color and edges, reflecting diverse features while also deepening the feature flow. This design is particularly important for detecting small-sized, low-resolution, and occluded road signs, as it preserves contextual information while capturing detailed features.

#### 2.2.2. Improved Neck Network

As illustrated in Figure 1, in the original YOLO11n model, the Neck network adopts a multi-scale feature fusion structure based on the FPN-PAN paradigm. Within the PAN structure, feature downsampling and cross-level fusion are achieved through successive convolutional layers with a stride of 2.

Although this structure successfully improves high-level semantics for medium and large objects, its performance degrades in scenes densely populated with small-scale targets. Due to continuous convolutional downsampling, local information is gradually attenuated, making it difficult for the model to capture the texture details of small road signs.

Furthermore, the feature fusion in the original Neck structure emphasizes the consistency of contextual semantics, resulting in insufficient preservation of the spatial structure of small-scale targets. This causes small-scale targets to be easily lost in high-level feature maps, thereby compromising detection performance.

**Figure 4:**
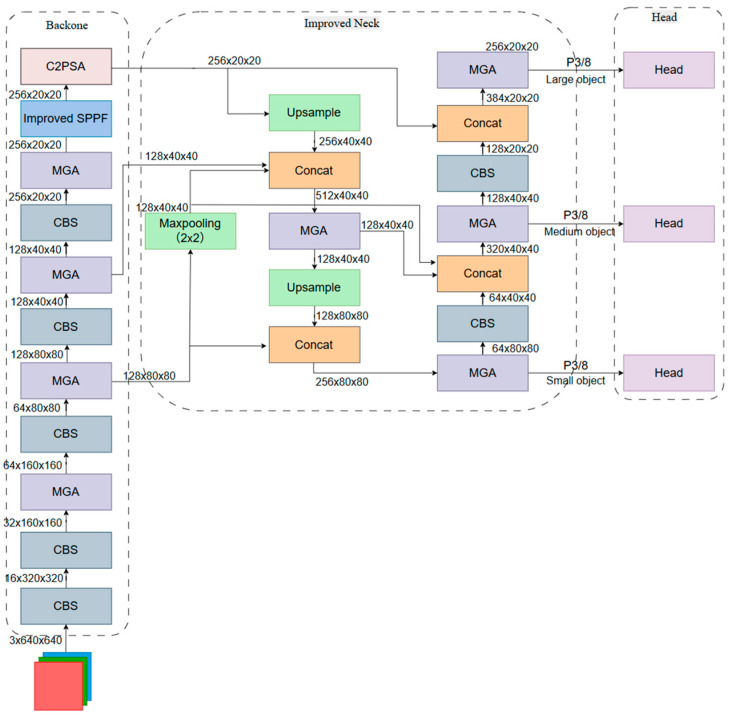
Architecture of improved Neck network.

To address the above issues, an improved Neck network (Figure 4) is proposed. This structure integrates the semantic expressiveness of convolutional downsampling with the spatial preservation capacity of pooling operations. Consequently, it enhances the retention and propagation of fine-grained local features for small targets.

Considering that the input road sign images in the network shown in Figure 4 are represented in the RGB color model, the dominant colors of road signs—for instance, the R channel in red prohibition signs and the B channel in blue mandatory signs—typically exhibit pronounced single-channel maximum responses relative to the other channels, whereas the edges, owing to abrupt color transitions, are often associated with local extrema across multiple channels. This channel-wise amplitude advantage at the input level is further reinforced by convolutional kernels during network training. The network learns filters that are sensitive to specific colors, allowing the sign regions to remain locally maximal responses on the corresponding feature maps [33]. Consequently, the max-pooling operation can effectively preserve these color-dominated discriminative peak features while suppressing background noise.

Based on the above analysis, the improved Neck network incorporates a new downsampling branch. The newly added branch originates from the MGA module in the 5th layer of the Backbone network. First, a max-pooling operation with a stride of 2 is applied to the output of this MGA module, transforming the feature map from 128×80×80 to 128×40×40. This output serves as input to the first Concat layer in the FPN and PAN structures of the neck network.

The max-pooling operation effectively preserves local extreme responses and exhibits stronger sensitivity to high-contrast regions and edge information. This structure provides an upsampling and downsampling path that is more conducive to preserving small-scale structural information for the Neck without introducing additional computational burden, forming a multi-path feature expression mechanism that is particularly suitable for road sign detection tasks dominated by small targets.

#### 2.2.3. Improved SPPF Module

**Figure 5:**
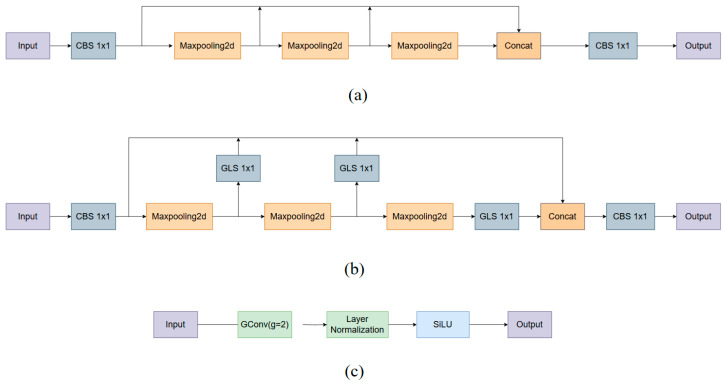
Architecture of the improved SPPF module. (**a**) SPPF; (**b**) Improved SPPF; (**c**) GLS 1 × 1.

As shown in Figure 5a, the SPPF module is located at the end of the Backbone network in YOLO11n. After the image undergoes 32× downsampling, it assumes the core function of multi-scale feature aggregation. Through fusing the max-pooling outputs at different scales, the module effectively strengthens global feature characterization, which is particularly well-adapted for road sign detection tasks involving targets with prominent shape features.

Nevertheless, in driving scenarios, road signs occupy a relatively small spatial proportion compared to background elements such as roads, vehicles, and trees. Coupled with the degraded color contrast under low-resolution or dim lighting, features tend to become highly sparse following 32× downsampling, which inevitably results in losing essential information.

To fully exploit the representation potential of sparse features in the SPPF module, an improvement is made to the original structure, as illustrated in Figure 5b. A GLS module was introduced at the corresponding position to facilitate multi-channel information fusion.

In the improved SPPF module shown in Figure 5b, the input feature map first passes through a 1 × 1 CBS convolution layer, which performs channel fusion, batch normalization, and SiLU activation on all channels. This process strengthens the feature representation capability, improving the network’s training stability. The output of the 1 × 1 CBS convolution module is then passed through three cascaded max-pooling downsampling layers. The output of each pooling layer is further processed by a 1 × 1 GLS module.

As illustrated in Figure 5c, the theoretical design of the GLS 1 × 1 module is mainly reflected in the following two aspects:

Firstly, to address feature sparsity and channel redundancy, the 1 × 1 GLS module adopts Group Convolution (g = 2). Compared with standard convolution, group convolution forces the network to learn decoupled feature representations within specific channel groups. For example, shape-contour features can be separated from color-texture features. This hard constraint effectively prevents dominant background features in deep networks from suppressing weak traffic sign features. It significantly improves the fusion efficiency of multi-channel spatial information without increasing computational overhead.

Secondly, to address the pain point of highly unstable responses to small targets, the GLS module introduces Layer Normalization (LN) in place of the traditional Batch Normalization (BN). Because road sign features become extremely sparse after 32× downsampling, conventional BN is highly susceptible to variations across input samples. As a result, the representation at the feature level may fluctuate dramatically. This strong dependence on batch size makes it difficult for the model to maintain stable mapping relationships when handling deep sparse targets, thereby weakening the network’s ability to capture critical semantic information. Layer Normalization, on the other hand, focuses on normalizing the channel dimension of a single sample, completely eliminating the statistical dependence on batch size. This characteristic enables the GLS module to independently and accurately amplify the feature response of each sparse target at a specific scale. After Layer Normalization stabilizes the feature distribution, the features are further activated by the SiLU function. As a result, the network can still robustly highlight the local contextual information of traffic sign regions under complex road scenes and severely constrained computational conditions.

In summary, in the improved SPPF structure, the 1 × 1 GLS module is embedded between the stages of multi-scale pooling and feature fusion. It is not a simple structural stacking. Instead, through decoupling by groups and channel normalization, it performs high-quality semantic alignment and feature refinement on sparse responses under different receptive fields before feature concatenation. This design establishes a solid foundation for final high-precision detection.

## 3. Construction of Datasets

Two datasets were employed in the experiments. One was collected from the publicly available Roboflow Universe platform and the other was the CCTSDB2021 traffic sign dataset [34]. In terms of sample characteristics, the former is more aligned with the objectives of this study. The latter, by contrast, serves as a widely used reference dataset.

**Figure 6:**
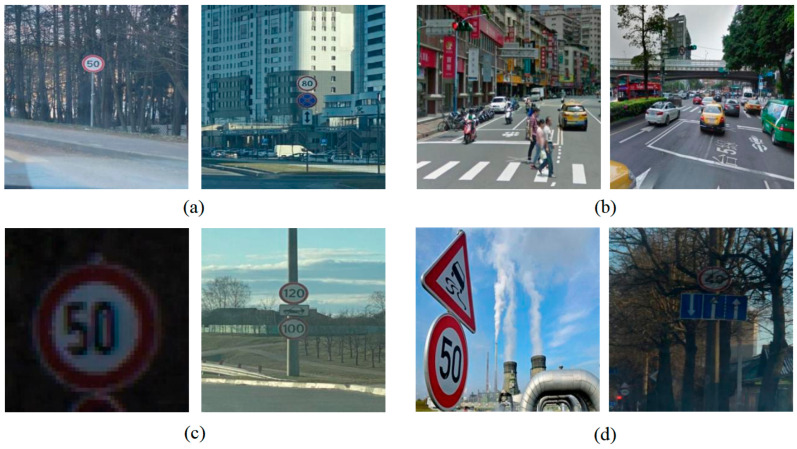
Four types of typical road sign images. (**a**) Single and Multiple Small Targets; (**b**) Complex Background; (**c**) Backlighting; (**d**) Tilt and Occlusion.

The dataset constructed from the publicly available Roboflow Universe source contains 15 categories of traffic signs, including traffic lights, no-parking signs, and speed limit signs with different speed limits. It includes 4969 images in total, among which 756 images contain more than two types of traffic signs. Overall, the dataset comprises 6012 traffic signs. This dataset was constructed by comprehensively incorporating typical road conditions such as small objects, complex backgrounds, backlighting, object tilt, and occlusion, which can fully cover various road conditions in real life and exhibits strong scene representativeness and wide recognition. Figure 6 shows sample images from the publicly available Roboflow Universe dataset.

As shown in Figure 6, the dataset incorporates diverse extreme road conditions to assess the model’s perception capability under non-ideal conditions. In particular, small objects occupy only a very limited proportion of pixels, thereby imposing stringent requirements on the deep feature perception capability of the feature extraction network. In contrast, backlighting and occlusion directly simulate the visual disturbances that most commonly lead to missed detections in real-world driving scenarios. Samples collected from such complex scenarios not only enrich the diversity of the dataset but also more effectively probe the perceptual limits of the model near the boundaries of recognition. This provides strong support for a comprehensive evaluation of model robustness.

Detailed information on the dataset is provided in Table 1.

CCTSDB2021, a widely used benchmark dataset for traffic sign detection, consists of 17,856 high-resolution images collected from real urban and highway environments, containing more than 40,000 annotated traffic sign instances in total. All traffic signs are categorized into three standard categories, namely, mandatory, prohibitory, and warning signs. This further facilitates the evaluation of the generalization capability and stability of the improved model.

## 4. Experiments

### 4.1. Dataset Processing

The selected road sign dataset covers road signs under different weather conditions, scales, types, and times, with all images resized to 640 × 640 pixels.

To ensure the effectiveness of model training and evaluation, the selected traffic sign dataset from the publicly available Roboflow Universe platform was strictly divided in a 7:2:1 ratio: 3530 images were used for training, 801 for validation, and 638 for testing. This dataset splitting method ensures a reasonable ratio of training, validation, and test sets, providing sufficient training samples for the model and guaranteeing the fairness and objectivity of the evaluation process. Consequently, this data processing solidifies the experiment’s reliability and validity, providing a robust groundwork for subsequent experiments [35].

To ensure fair and reproducible comparisons with existing studies, the CCTSDB2021 dataset was used according to its official split, which consists of 16,356 training images and 1500 test images. To rigorously evaluate model robustness, the test set covers diverse challenging illumination and weather conditions, including cloudy, nighttime, rainy, and foggy scenarios. This provides an ideal benchmark for evaluating the model’s generalization capability in small-object detection and adverse environments.

### 4.2. Experimental Environment

The experiments used an NVIDIA GeForce RTX 4050 GPU and an AMD Ryzen 7 7735H CPU with Radeon Graphics @ 3.20GHz CPU for model training and testing, with 16 GB of memory. Furthermore, the program was run using mainstream deep learning frameworks, including CUDA 12.6, Python 3.12.11, and PyTorch 2.8.0 + cu126.

The network parameter initialization configuration is shown in Table 2.

To investigate the convergence of the model during training, training was conducted for 100, 150, and 200 epochs respectively to optimize the object detection results.

### 4.3. Evaluation Metrics

To more comprehensively analyze the model’s performance, the experiments used the mean Average Precision (mAP), Frames Per Second (FPS), Parameters (Params), Precision, and Recall to evaluate the model’s performance.

Average precision (AP), obtained by integrating the Precision-Recall curve, is used to evaluate the overall performance of the detection model across different threshold ranges. A higher AP value indicates better accuracy and stability of the model in object detection. The calculation formula is shown in Equation (2).

mAP represents the arithmetic mean of AP values for all categories and is used to reflect the overall performance of the model in multi-category object detection tasks. Its calculation formula is shown in Equation (3).

(2) $$\mathrm{AP}=\int_{0}^{1}P(r)dr$$

(3) $$\mathrm{mAP}=\frac{\sum_{i=1}^{N}AP_{i}}{N}$$

where r represents the value of Recall, P(r) represents the precision at Recall r, and N represents the total number of samples.

FPS represents the number of image frames that the model can process per second and is a key metric for evaluating inference speed and real-time performance. A higher FPS value indicates faster inference speed and better real-time performance, enabling the model to complete image detection in a shorter time.

Params is a key metric for evaluating the complexity of a deep learning model. It is determined by the total number of trainable weights and biases. Although a larger number of parameters can improve the model’s representation capacity, it also leads to higher demands on computational resources and training time.

Precision represents the proportion of samples that the model predicted to be positive that actually turned out to be positive. It is used to measure the accuracy of the prediction results. The calculation formula is as follows:

(4) $$\mathrm{Precission}=\frac{TP}{TP+FP}$$

where TP represents the number of positive samples correctly classified as positive and FP denotes the count of negative samples misclassified as positive.

In autonomous driving scenarios, recall is a critical metric with direct implications for life safety [36]. Recall represents the proportion of all actual positive samples that are correctly identified by the model.

(5) $$\mathrm{Recall}=\frac{TP}{TP+FN}$$

### 4.4. Comparative Experiments

To ensure the fairness and reproducibility of the comparison results, all models participating in the comparison were trained and evaluated under exactly the same experimental conditions. All hardware experiments were conducted on a unified setup comprising an NVIDIA GeForce RTX 4050 GPU and an AMD Ryzen 7 7735H CPU, with the random seed of the deep learning framework fixed to eliminate stochastic fluctuations during the training process. The dataset was partitioned with identical training, validation, and test splits at a strict ratio of 7:2:1, and exactly the same data augmentation strategy, including Mosaic and MixUp operations, was applied. All models were trained from scratch without loading any pretrained weights. All hyperparameters were kept consistent across experiments: the stochastic gradient descent (SGD) optimizer was employed with a momentum of 0.937 and a weight decay of 0.0005; the initial learning rate was set to 0.01, the batch size to 16, and the input image resolution was uniformly fixed at 640 × 640 pixels. All models were trained for 200 epochs, and the CIoU (Complete Intersection over Union) loss function was uniformly adopted. During the evaluation phase, all models used the same confidence threshold and IoU threshold and comprehensively considered metrics such as mAP@0.5, mAP@0.5–0.95, recall, FPS, and number of parameters to ensure the comprehensiveness and fairness of the comparison.

To explicitly demonstrate the superiority of the improved model, classic models including YOLO26n, YOLO12n, YOLO11n, YOLO10n, YOLO8n, YOLO6n, YOLO5n and RT-DETR were selected for comparative experiments. Additionally, the experiments incorporated two-stage detection models, such as Faster R-CNN, as well as other representative novel road sign detection methods proposed by researchers. The experimental results are shown in Table 3.

In autonomous driving scenarios, the recall of traffic sign detection has direct implications for life safety. Missing even a single “Stop” or “Speed Limit” sign may result in severe accidents. Therefore, priority should be given to high recall when selecting the baseline model. According to the recall results reported in Table 3, RT-DETR (92.28%), YOLO5n (91.79%), YOLO26n (90.44%), and YOLO12n (88.91%) all outperform YOLO11n (88.77%) in terms of recall. RT-DETR achieves the highest recall among all compared models. However, its parameter count reaches 32.01 M, and its FPS is only 38. Such performance is insufficient to satisfy the stringent requirements of autonomous driving for real-time inference and embedded deployment. YOLO5n achieves a recall of 91.79%, operates at 202 FPS, and contains 2.50 M parameters. Its overall performance is well balanced, which can make it seem an attractive choice for the baseline model. However, YOLO5n is still built on an older architecture with anchor-based prediction and a coupled detection head. Moreover, it lacks a native attention mechanism. Consequently, its performance upper bound is clearly lower than that of YOLO11n. By contrast, YOLO11n adopts a more advanced design with an anchor-free paradigm, a decoupled head, and the C2PSA attention module. Such an architecture provides greater room for further optimization, particularly in addressing the channel sparsity of traffic sign features. YOLO26n achieves a recall of 90.44%, which is higher than that of YOLO11n. However, its inference speed drops to 114 FPS, far below the 193 FPS of YOLO11n. In high-speed autonomous driving scenarios, lower inference speed results in greater processing latency. This may directly delay decision-making and thus pose safety risks. YOLO12n exhibits a similar pattern. Although its recall reaches 88.91%, slightly higher than the 88.77% of YOLO11n, its inference speed is only 153 FPS, which still remains below that of YOLO11n. Moreover, its mAP@0.5–0.95 is 82.46%, noticeably lower than the 83.17% achieved by YOLO11n.

Comprehensively evaluating the public datasets from Roboflow Universe, YOLO11n delivers the highest inference speed (193 FPS) and competitive overall accuracy (83.17% mAP@0.5–0.95), while achieving recall rates comparable to those of YOLO12n and YOLO26n, lagging by only 0.14 and 1.67 percentage points, respectively. A higher inference speed not only satisfies the requirement for real-time performance but also leaves sufficient computational budget for more sophisticated preprocessing and postprocessing strategies. In addition, as a more recent lightweight model, YOLO11n offers greater room for further architectural optimization. Therefore, YOLO11n was selected as the baseline model, with recall improvement set as the primary objective of subsequent optimization.

Next, in comparison with existing improved road sign detection models (including Improved YOLO8s and Improved YOLO5s), the superiority of the improved YOLO11n model can be more clearly demonstrated. Experimental results show that the improved YOLO11n model outperforms the above methods in terms of Precision, Recall and mAP. For example, although the Improved YOLO8n model achieves a slightly higher mAP value than our model, its Precision, Recall, and FPS are significantly lower. The accuracy of the improved YOLO5s is 0.41% higher than that of the improved YOLO11n model, but it is lower in all other metrics. In terms of parameter count, the proposed model contains only 4.35 M parameters. This is about 70% and 40% fewer than Improved YOLO8s (14.60 M) and Improved YOLO5s (7.20 M), respectively. The model therefore achieves a substantial reduction in complexity while maintaining high detection accuracy. As a result, it is better suited for deployment on resource-constrained embedded devices. The results demonstrate that the improved YOLO11n model further optimizes real-time performance while maintaining detection accuracy, making it more suitable for real-time road sign detection tasks in complex traffic scenarios.

Overall, the improved YOLO11n model (denoted as Ours in Table 3) achieves a recall of 92.94%, which is a core safety-critical metric. This represents an improvement of 4.17 percentage points over the baseline YOLO11n (88.77%). Moreover, it surpasses all compared models, thereby markedly reducing the potential driving risks associated with missed detections. Meanwhile, the improved model maintains a real-time inference speed of 134 FPS and a lightweight parameter count of 4.35 M. This indicates that the improvement in recall is achieved without sacrificing real-time performance or deployment feasibility.

To evaluate the stability and reliability of the improved YOLO11n model, multiple training runs were conducted using random seeds ranging from 0 to 5. The mean, standard deviation, and 95% confidence interval were then calculated for each key metric to evaluate performance consistency. On average, the model achieved mAP@0.5 of 96.96% (±0.11), precision of 83.94% (±0.20), recall of 92.94% (±0.39), and accuracy of 98.46% (±0.20). The limited variation in these results suggests that the model maintains strong stability across different initialization conditions.

In addition, a paired t-test was performed to compare the improved model with the baseline model trained using seed 0. The results indicate that the improvements in mAP@0.5 (t = −0.38, *p* = 0.63) and mAP@0.5–0.95 (t = 0.25, *p* = 0.80) are not statistically significant. By contrast, the gains in accuracy (t = −2.75, *p* = 0.029) and recall (t = −0.32, *p* = 0.009) are statistically significant, indicating that the proposed model achieves meaningful improvements. These results confirm that our model not only maintains strong consistency but also provides genuine performance enhancement.

To further assess the performance of the model on a large-scale dataset, YOLO12n, YOLO11n, YOLO10n, YOLO8n, Faster R-CNN and the improved YOLO11n were trained and tested on the CCTSDB2021 dataset. The results are presented in Table 4.

The experimental results indicate that the improved YOLO11n achieves the best balance of detection accuracy and overall performance. Its recall reaches 75.05%, which is markedly higher than that of all competing models, indicating a clear advantage in reducing missed detections. Meanwhile, its mAP@0.5 and mAP@0.5–0.95 reach 81.28% and 52.29%, respectively, both of which are the highest among all models. These results demonstrate that the proposed improvements effectively enhance detection accuracy and localization robustness under different IoU thresholds. Although the precision (P) of Ours is 87.17%, which is slightly lower than that of YOLO11n and YOLO10n, it achieves a more balanced and overall superior detection performance. In terms of efficiency, Ours achieves 134 FPS. Although this is lower than the 193 FPS of YOLO11n and the 221 FPS of YOLO10n, it still satisfies the requirement for real-time detection. Its parameter count is 4.35 M, which is slightly higher than that of the baseline YOLO11n. This indicates that the gain in accuracy is accompanied by a moderate increase in model complexity, while remaining within a reasonable range.

In summary, the improved YOLO11n substantially improves recall and multi-scale localization accuracy while maintaining strong real-time performance. Its overall performance surpasses that of the original YOLO11n as well as the other comparison models. These results validate the effectiveness and generalization capability of the proposed method on a large-scale dataset.

### 4.5. Ablation Experiment

Compared to the original YOLO11n model, the proposed model introduces the MGA module, a Neck network structure, and an improved SPPF module. To verify the efficacy of the improved modules and structure, the following four sets of ablation experiments were designed:

(1) The Original YOLO11n network.
(2) Scheme (1) combined with the MGA module.
(3) Scheme (1) combined with the improved Neck network.
(4) Scheme (1) combined with the improved SPPF module.
(5) Scheme (1) combined with all improvements, including the MGA module, Neck network, and optimized SPPF module.

The experimental environment remains the same as described in Section 4.2, and the detailed experimental procedure is shown in Table 5.

As indicated by the ablation results in Table 5, the comprehensive performance of the model exhibits a clear improvement trend as the MGA module, the improved neck, and the improved SPPF module are progressively introduced into YOLO11n.

In terms of the effect of individual modifications, Experiment II (YOLO11n + MGA) achieves the most pronounced gain in recall (R), reaching 92.31%, which is 3.54 percentage points higher than the baseline. Its mAP@0.5 also increases to 96.24%, indicating that this combination is particularly effective in improving detection completeness. Experiment III (YOLO11n + Improved Neck) likewise delivers concurrent gains in both recall and mAP. By contrast, Experiment IV (YOLO11n + Improved SPPF) places greater emphasis on precision (P), which rises to 97.01%, although recall decreases slightly.

With all proposed improvements incorporated, Experiment V attains the best recall (92.94%) and mAP@0.5 (96.96%). These values are 4.17 and 1.42 percentage points higher than those of the baseline, respectively. Although its precision (96.13%) is 0.11 percentage points lower than that of the baseline, the gain in recall clearly outweighs the loss in precision. In terms of inference speed, Experiment V achieves 134 FPS. Although this is lower than the 193 FPS of the baseline, it still exceeds the typical threshold for real-time detection, which is usually around 30 FPS. Therefore, it remains suitable for practical deployment.

Considering the application scenario of traffic sign detection, the cost of false negatives is far greater than that of false positives. Missing critical signs, such as stop signs or speed limit signs, may cause the vehicle to miss essential traffic information and thus lead to safety hazards. False positives, by contrast, can be filtered and compensated for to some extent through subsequent decision-making logic. Therefore, as long as precision remains within an acceptable range, prioritizing recall improvement is well justified from both engineering and safety perspectives. Taken together, the improvement scheme in Experiment V provides a more favorable balance between detection completeness and real-time performance, and is therefore better suited to traffic sign detection.

**Figure 7:**
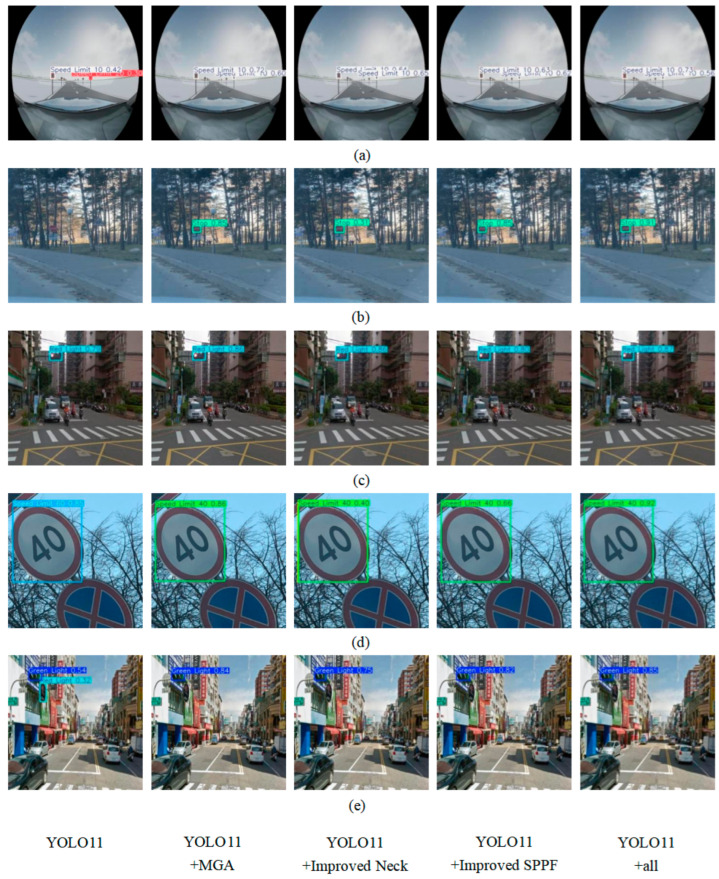
Visualization results of ablation experiments. (**a**) Long Distance; (**b**) Blur; (**c**) Low-Light Conditions; (**d**) Tilt; (**e**) Complex Backgrounds.

To illustrate the contribution of each improvement more intuitively, several typical images were selected for comparison, and the comparison results are shown in Figure 7.

Across four typical road scenarios (Figure 7a–e), the improved YOLO11n achieves marked improvements in road sign detection performance under conditions of long distance, blur, low light, tilt, and complex background.

In scenario a, the baseline YOLO11n model has difficulty detecting road signs in distant images, resulting in false detections (misidentifying a “Speed Limit 10” sign as “Speed Limit 20”). After introducing the designed MGA module, the detection effect of distant targets is significantly improved by enhancing multi-scale feature fusion. The improved Neck network adds a max-pooling layer after the output of the fifth layer MGA module in the backbone network, which alleviates the loss of small-scale identifier features. Furthermore, the improved SPPF module adds a 1 × 1 GLS layer after max-pooling, reducing background interference and enhancing global feature extraction, making the signs more prominent. Finally, the improved model effectively reduces false detections and achieves more accurate detection.

In scenario b, the basic YOLO11n model struggles with low-light environments, obscured objects, and blurry road signs, resulting in missed detections. The model with the MGA module successfully detected the “Stop” sign. The optimized Neck network enhances the ability to identify small targets by preserving detailed information. The enhanced SPPF module, with the introduction of a 1 × 1 GLS layer, further separates traffic signs from the background. This improves localization accuracy and robustness in complex environments.

In scenario d, road signs at multiple angles significantly impact model performance, and the baseline YOLO11n model struggles to detect them accurately under such conditions. The MGA module enhances spatial pose perception and markedly improves the model’s ability to capture features of targets under non-frontal viewpoints. The improved neck network further refines feature representations under complex poses by strengthening global feature fusion. Combined with the fine-grained feature extraction enabled by the improved SPPF module, the model is able to maintain highly robust detection performance even when traffic signs exhibit large variations in orientation.

In scenario e, the original YOLO11n model exhibits clear limitations in perception. It struggles to effectively separate extremely small traffic signs from cluttered backgrounds and is prone to falsely identifying background regions as traffic signs. The MGA module substantially enhances the discriminability of extracted features through lightweight convolutional feature enhancement and multi-scale feature fusion. The improved Neck network draws a branch from the Backbone network and performs multi-scale interaction operations through the max-pooling layer, which effectively preserves the characteristics of small-scale targets. In addition, the improved SPPF module improves global information extraction and improves the feature representation of road signs under complex road backgrounds.

In summary, the gradual integration of these improved modules significantly enhances the target detection performance of the YOLO11n model in complex road scenarios, especially under adverse conditions such as long distance, blur, low light, and tilted signs. Ultimately, the improved model demonstrates more accurate, stable, and real-time road sign detection performance.

To explore the impact of the GLS module on the multi-scale feature extraction capability of the improved SPPF module, a series of experiments were conducted by adjusting the number of groups in the Group Convolution module. Table 6 presents the experimental results with relatively favorable performance under different group numbers.

The experimental results demonstrate that the model achieves optimal comprehensive performance when Group = 2. In terms of computational cost, as the number of groups increases from 1 to 32, both the model’s parameters and Floating-point Operations Per Second (FLOPs) exhibit a gradual decline and eventually stabilize. Specifically, the configuration with Group = 2 features 4.410 M parameters and 7.016 Giga Floating-point Operations Per Second (GFLOPs), maintaining moderate model complexity while ensuring computational efficiency. Regarding detection precision, Group = 2 outperforms all other groups, reaching an mAP@0.5 of 96.96% and an mAP@0.5–0.95 of 83.94%. These results fully indicate that the GLS module under this configuration can effectively enhance the multi-scale feature extraction capability of SPPF, thereby improving the model’s recognition performance in complex road sign scenarios.

Nonetheless, excessively increasing the number of groups leads to performance degradation. When the number of groups is increased to 8, 16, and 32, although the parameter count and computational cost are further reduced, both accuracy metrics of the model exhibit varying degrees of decline, with the drop in mAP@0.5 being particularly pronounced. This demonstrates that an excessively large group number is prone to disrupting effective feature fusion, thereby impairing multi-scale representation and ultimately deteriorating detection accuracy.

In summary, when taking into account detection accuracy, model complexity, and computational efficiency collectively, Group = 2 emerges as the optimal configuration for the GLS module. This configuration effectively strengthens the feature extraction capability of the SPPF module while reducing the model’s computational cost, making it more adaptable to real-time road sign detection in complex traffic environments.

## 5. Conclusions

To address the challenges posed by complex backgrounds, multi-scale targets, long-distance scenarios, and low-light conditions in driving environments, this paper proposes a traffic sign detection method based on an improved YOLO11n. By designing an MGA feature extraction module, enhancing the multi-scale feature fusion capability of the neck network, and strengthening the global information extraction ability of the SPPF module, the proposed model improves the detection of low-resolution, occluded, and small traffic signs. It also exhibits stronger robustness across diverse traffic environments.

The experimental results indicate that the improved model achieves gains of 1.42% and 0.77% in mAP@0.5 and mAP@0.5:0.95, respectively. More importantly, recall is improved by 4.17%, which is critical for ensuring decision safety in autonomous driving systems. Meanwhile, the model reaches 134 FPS, satisfying the requirement for real-time detection. These results demonstrate its accuracy and robustness in complex environments.

Although the improved model achieves a good balance between accuracy and real-time performance, there remains a certain gap from ideal performance. For instance, simultaneously detecting multiple extremely small traffic signs in long-distance imaging scenarios is still challenging. Future work will focus on optimizing the network architecture to improve the model’s adaptability to small objects. Further efforts will also be devoted to advancing lightweight design while maintaining high detection accuracy and real-time performance under hardware-constrained conditions.

## Figures and Tables

**Table 1:** Statistics on the categories and distribution of road sign datasets.

Label	Number
Green Light	774
Red Light	787
Speed Limit 10	22
Speed Limit 20	387
Speed Limit 30	468
Speed Limit 40	343
Speed Limit 50	404
Speed Limit 60	422
Speed Limit 70	449
Speed Limit 80	440
Speed Limit 90	240
Speed Limit 100	365
Speed Limit 110	139
Speed Limit 120	356
Stop	416

**Table 2:** Experimental environment details.

Parameter	Value
Optimizer	SGD (momentum = 0.937, weight decay = 0.0005)
Batch_size	16
Iterations	343,313
Initial Learning Rate	0.01
Loss function	CloU

**Table 3:** Performance Comparison of Different Models on the Public Roboflow Universe Dataset.

Experiment No.	Model	P (%)	R (%)	mAP@0.5 (%)	mAP@0.5–0.95 (%)	FPS	Params (M)
1	Faster R-CNN [37]	84.40	87.60	86.35	70.74	22	41.81
2	YOLO26n [38]	96.07	90.44	95.95	82.63	113	2.38
3	YOLO12n [39]	95.60	88.91	95.50	82.46	153	2.56
4	YOLO11n [22]	96.24	88.77	95.54	83.17	193	2.58
5	YOLO10n [28]	92.64	88.07	94.59	82.05	221	2.26
6	YOLO8n [1]	94.77	91.14	95.78	83.13	200	3.00
7	YOLO6n [40]	94.76	86.61	94.07	81.70	211	4.23
8	YOLO5n [6]	95.24	91.79	96.18	83.31	202	2.50
9	RT-DETR [41]	93.41	92.28	94.76	81.95	38	32.01
10	Improved YOLO8s [42]	95.30	90.26	97.01	83.97	69	10.13
11	Improved YOLO5s [43]	96.54	91.52	96.55	82.83	82	11.23
12	Ours	96.13	92.94	96.96	83.94	134	4.35

**Table 4:** Performance comparison of different models on the CCTSDB2021 dataset.

Experiment No.	Model	P (%)	R (%)	mAP@0.5 (%)	mAP@0.5–0.95 (%)	FPS	Params (M)
1	YOLO12n [39]	84.81	68.45	75.40	47.91	153	2.56
2	YOLO11n [22]	88.42	72.53	79.87	51.62	193	2.58
3	YOLO10n [28]	88.32	71.65	80.70	51.42	221	2.26
4	YOLO8n [1]	86.00	72.12	79.41	50.53	200	3.00
5	YOLO5n [6]	80.80	73.90	80.31	50.73	202	2.50
6	Faster R-CNN [37]	60.73	49.86	51.93	39.25	22	41.81
7	Ours	87.17	75.05	81.28	52.29	134	4.35

**Table 5:** Ablation study analysis of the improved YOLO11n model.

Network	MGA	Improved Neck	Improved SPPF	P (%)	R (%)	mAP@0.5 (%)	mAP@ 0.5–0.95 (%)	FPS
YOLO11n				96.24	88.77	95.54	83.17	193
√	√			95.16	92.31	96.24	83.91	145
√		√		95.12	90.73	96.16	83.11	196
√			√	97.01	87.33	95.67	82.87	192
√	√	√	√	96.13	92.94	96.96	83.94	134

**Table 6:** Experimental results with different group numbers in group convolution.

Group	Params (M)	P (%)	R (%)	mAP@ 0.5 (%)	mAP@ 0.5–0.95 (%)	GFLOPS
1	4.410	95.12	93.31	96.47	83.77	7.056
2	4.410	96.13	92.94	96.96	83.94	7.016
8	4.397	93.46	91.47	96.13	83.21	6.987
16	4.395	95.98	90.65	96.35	84.16	6.982
32	4.394	95.97	92.77	96.48	83.58	6.980

## Data Availability

The dataset sourced from the Roboflow Universe platform and the CCTSDB2021 dataset can be accessed publicly at the following URLs: https://universe.roboflow.com/selfdriving-car-qtywx/self-driving-cars-lfjou/dataset (accessed on 12 April 2026) and https://github.com/csust7zhangjm/CCTSDB2021 (accessed on 13 April 2026).

## Abbreviations

The following abbreviations are used in this manuscript:

YOLO	You Only Look Once
MGA	Multi-path Gated Aggregation
SPPF	Spatial Pyramid Pooling—Fast
GLS	Group Convolution-Layer Normalization-SiLU
mAP	Mean Average Precision
FPS	Frames per second
RBF	Radial Basis Function
RANSAC	Random Sample Consensus
Faster R-CNN	Faster Region-based Convolutional Neural Network
CNN	Convolutional Neural Network
DWConv	Depth-Wise Separable Convolution
PAN	Path Aggregation Network
FPN	Feature Pyramid Network
Conv	Convolution
CBS	Convolution-Batch Normalization-SiLU
C3K2	CSP Stage Block with 3 Convolutions and 2-input Bottleneck
C2f	CSP Stage Block with 2-flows
C2PSA	Cross Stage Partial with Pyramid Squeeze Attention
C3K	CSP Stage Block with 3 Convolutions
AP	Average precision
GFLOPS	Giga Floating-point Operations Per Second
FLOPS	Floating-point Operations Per Second
Params	Parameters
